# Hsa_circ_0079662 induces the resistance mechanism of the chemotherapy drug oxaliplatin through the TNF‐α pathway in human colon cancer

**DOI:** 10.1111/jcmm.15122

**Published:** 2020-04-03

**Authors:** Mingfen Lai, Guiju Liu, Ruijun Li, Hua Bai, Jizhi Zhao, Peng Xiao, Jiazhuan Mei

**Affiliations:** ^1^ Department of Oncology The Second Clinical Medical School of Southern Medical University Guangzhou China; ^2^ Department of Oncology Zhengzhou People's Hospital Affiliated to Southern Medical University Zhengzhou China

**Keywords:** ceRNA, colorectal cancer, HOXA9, Hsa_circ_0079662, TNF‐α pathway

## Abstract

The aim of the study was to research the biological functions of circRNA (hsa_circ_0079662) and its underlying mechanism in colorectal cancer. Drug‐resistant cell lines (HT29‐LOHP, HCT116‐LOHP, HCT8‐LOHP) were separately dealt with oxaliplatin concentration gradient (0.1‐10 μmol/L). Real‐time PCR, Western blotting, dual‐luciferase assay, miRNA pull‐down assay, coimmunoprecipitation and ELASA were performed to explore the mechanism of chemotherapy drug oxaliplatin resistance in CRC. The results showed that the expression of hsa_circ_0079662 was increased in drug‐resistant cell lines by RT‐PCR. The expression of HOXA9, TRIP6, Vcam‐1, VEGFC, MMP3, MMP9 and MMP14 was higher by Western blotting. Interaction between HOXA9 and TRIP6 in CO‐IP detection. Additionally, the cytokines TNF‐α, IL‐1 and IL‐6 were also found. In conclusion, hsa_circ_0079662, as a ceRNA binding with hsa‐mir‐324‐5p, can regulate target gene HOXA9 and induced the mechanism of chemotherapy drug oxaliplatin resistance in CRC through the TNF‐α pathway in human colon cancer.

## 1 INTRODUCTION

Colorectal cancer (CRC) is the common gastrointestinal malignancy that occurs in the colon.^1^, ^2^, ^3^, ^4^ The incidence of gastrointestinal tumours accounted for the third in cancer. Its gross form is polypoid and ulcerative. CRC can be circumferential development along alvine wall, along alvine canal longitudinal diameter up and down spread or to alvine wall depth infiltrate, besides classics lymphatic canal, blood stream is transferred and outside local encroach, still can be planted inside peritoneal cavity or along suture, incision face diffusion is transferred.^2^, ^3^

The existence of covalently closed circular RNAs (circRNAs) is observed by electron microscopy almost 40 years.^5^, ^6^, ^7^, ^8^, ^9^ CircRNAs are successfully identified across various species by high‐throughput sequencing and bioinformatic analysis.^10^, ^11^, ^12^, ^13^, ^14^ Exon skipping and direct back splicing are the two mechanisms to format exonic or exon‐intron circRNAs with no polyadenylated tail, and can be regulated by some splice factors.^15^, ^16^, ^17^, ^18^, ^19^ Salient features of circRNAs include remarkable stability, high abundance, evolutionary conservation.^10^, ^11^, ^12^, ^20^ Besides, certain circRNAs are considerably more abundant than cognate mRNAs.^11^, ^12^, ^13^ Several studies found that circRNAs took part in gene expression.^19^, ^21^, ^22^, ^23^, ^24^ Recently, circRNAs were reported function as ‘miRNA sponges’ and could make a negative regulation on miRNAs.^10^, ^14^, ^25^ CDR1as or ciRS‐7,^10^, ^14^ well‐known circRNA, which harbours up to 74 canonical binding sites for miR‐7, abundantly sequesters miR‐7 away from CDR1, and makes it become an effective ‘miR‐7 sponges’.

HOXA9 raw letter analysis in colon cancer tissues expression abundance is high, the HOXA9 of protein expression in endothelial cells, can promote under inflammatory cytokines induce tissue adhesion, the function of inducing angiogenesis, promoting the characteristics of the tumour metastasis, therefore, and prone to metastasis after colon cancer drug resistance of characterization, so choose the gene as the research object. According to database analysis, the corresponding circRNAs of HOXA9 are hsa_circ_0079662, then the related miRNAs are obtained from circbank and circinteractome, and then the miRNA bound to HOXA9 is obtained from miWalk database. The intersection of these miRNA is obtained from the Vein diagram, and hsa‐mir‐324‐5p can be bound to hsa_circ_0079662 and HOXA9, to explore the regulatory effect of this circRNA as ceRNA on CRC after drug resistance.

## 2 MATERIALS AND METHODS

### 2.1 Cell culture

HT29, HCT116 and HCT8 were purchased from the Cell Bank of the Chinese Academy of Sciences. Cells were maintained in medium containing 10% FBS in a 37°C incubator with 5% CO_2_.^26^ Oxaliplatin concentration gradient was used to induce drug resistance in cells and drug‐resistant cell lines (HT29‐LOHP, HCT116‐LOHP and HCT8‐LOHP) were obtained, numbered A1, A2 and A3, respectively, while colon cancer cell lines were numbered B1, B2 and B3.

### 2.2 RT‐PCR

RNA was extracted with Trizol reagent, and cDNA was synthesized using the NCode TM miRNA First‐Strand cDNA Synthesis Kit.^27^, ^28^ RT‐PCR was performed by the following conditions: 95°C, 10 minutes; 95°C, 30 seconds, 60°C, 2 minutes. The expression was calculated by the 2^−ΔΔCt^.

### 2.3 Fluorescence in situ hybridization analysis

Slides were treated with 0.2 mol/L HCl, washed for 5 minutes, incubated in 4% pepsase for 10 minutes and washed for 5 minutes. The slides were prehybridized with prehybridization for 2 hours, hybridization using probe was performed overnight at 37°C, and slides were rinsed with 0.3% NP‐40 for 30 minutes and counterstained with DAPI for 5 minutes. The images were acquired using a confocal microscope (Leica).

### 2.4 Flow cytometry

Cells were acquired by flow cytometry (FACScan, BD Biosciences) and analysed by FlowJo 7.6.1.

### 2.5 Transwell assay

About 1 × 10^5^ cells were added to 24‐well plates. Using a cotton swab, non‐migrated cells in the upper chamber were removed after migration, and the cotton filters were fixed with 4% paraformaldehyde.^29^ Cell number was counted in five random fields of each chamber under a microscope.

### 2.6 miRNA pull‐down assay

About 1 × 10^7^ cells were harvested, and the probe was incubated with C‐1 magnetic beads at 25°C. After washing, the RNA complexes bound to the beads were eluted and extracted with RNeasy Mini Kit for RT‐PCR. Biotinylated‐circRNA probe was designed and synthesized by RiboBio. In brief, stably expressed circRNA was transfected with biotinylated miRNA mimics or mutant (50 nmol/L) using Lipofectamine RNAiMax. About 50 μL of the cell lysates was aliquot for input. The remaining cell lysates were incubated with C‐1 magnetic beads at 4°C for 3 hours and then washed. The bound RNAs were purified using RNeasy Mini Kit for the analysis.^25^

### 2.7 Western blotting

Cells were prepared by lysis with proteinase inhibitor. The protein was quantified by BCA kit (Pierce Biotechnology, IL). Each sample was separated by 15% SDS‐PAGE, and the membranes were incubated for 12 hours at 4°C with antibodies and then incubated with goat‐anti‐mouse IgG (Santa Cruz Biotechnology). The signals were detected with ECL reagents.^29^

### 2.8 Luciferase reporter assay

A dual‐luciferase reporter assay kit (Promega) was used to determine the luciferase activity 48 hours after transfection. Cells were harvested 48 hours after transfection for luciferase assay using a luciferase assay kit according to the manufacturer's protocol. The values were normalized to those obtained for miRNA negative control transfection.

### 2.9 Coimmunoprecipitation (Co‐IP)

Pierce IP Lysis Buffer was used for cell lysates. Co‐IP was then performed using the PureProteome™ Protein G Magnetic Bead System. Normal rabbit lgG (Millipore) was used as a negative control antibody.

### 2.10 ELISA

Levels of TNF‐α, IL‐1 and IL‐6 in cell culture supernatants were measured using ELISA kit (Cyman system) for TNF‐α, IL‐1 and IL‐6 according to the instruction manual.

### 2.11 Xenograft models

The animal experiments in this study were approved and reviewed by the Animal Research Committee. The tumorigenesis of the cell lines identified in vitro experiment into nude mice by tail vein injection was the animal model of CRC and the animal model of CRC drug resistance. si‐hsa_circ_0079662 adenovirus was constructed and injected into each group of model mice via tail vein, numbered as A: si‐colon cancer model, B: si‐colon cancer drug resistance model, C: colon cancer model, D: colon cancer drug resistance model. Blood samples from each group of model animals were taken, and the apoptosis characteristics of each group of blood samples were analysed by flow analysis. The proliferation of blood samples in each group was detected by Brdu. RNA was extracted from colon cancer tissues of each group of animal models, and the expression of hsa‐mir‐324‐5p was detected by RT‐PCR. Proteins from colon cancer tissues of animal models were extracted, and the expression levels of proteins HOXA9, TRIP6, vcam‐1, VEGFC, MMP3, MMP9 and MMP14 were detected by Western blot. Interaction between HOXA9 and TRIP6 in CO‐IP detection. The expression levels of cytokines TNF‐α, IL‐1 and IL‐6 in the blood samples of each group of model animals were detected by ELISA.

### 2.12 Statistical analysis

Results were performed using SPSS 19.0^27^ and presented as the mean ± SD from three independent experiments. A value of *P* < .05 was considered to indicate a statistically significant difference.

## 3 RESULTS

### 3.1 Hsa_circ_0079662 is up‐regulated in drug‐resistant CRC cell lines

**Figure 1:**
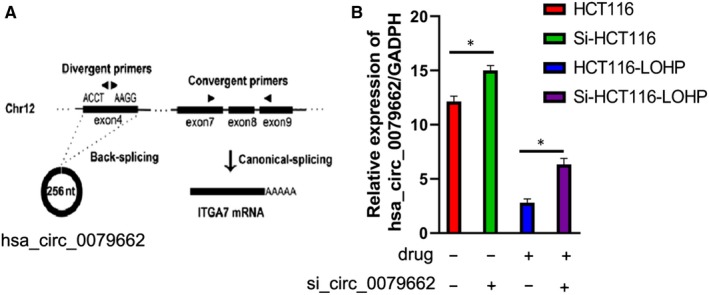
Hsa_circ_0079662 is up‐regulated in drug‐resistant CRC cell lines

To investigate the expression of hsa_circ_0079662 in CRC cells, RT‐PCR was conducted to examine its expression in CRC cells. Results revealed that hsa_circ_0079662 levels were significantly higher than that of in B1, B2 and B3 (*P* < .05) (Figure 1).

### 3.2 Hsa_circ_0079662 promotes growth of drug‐resistant CRC cell lines

**Figure 2:**
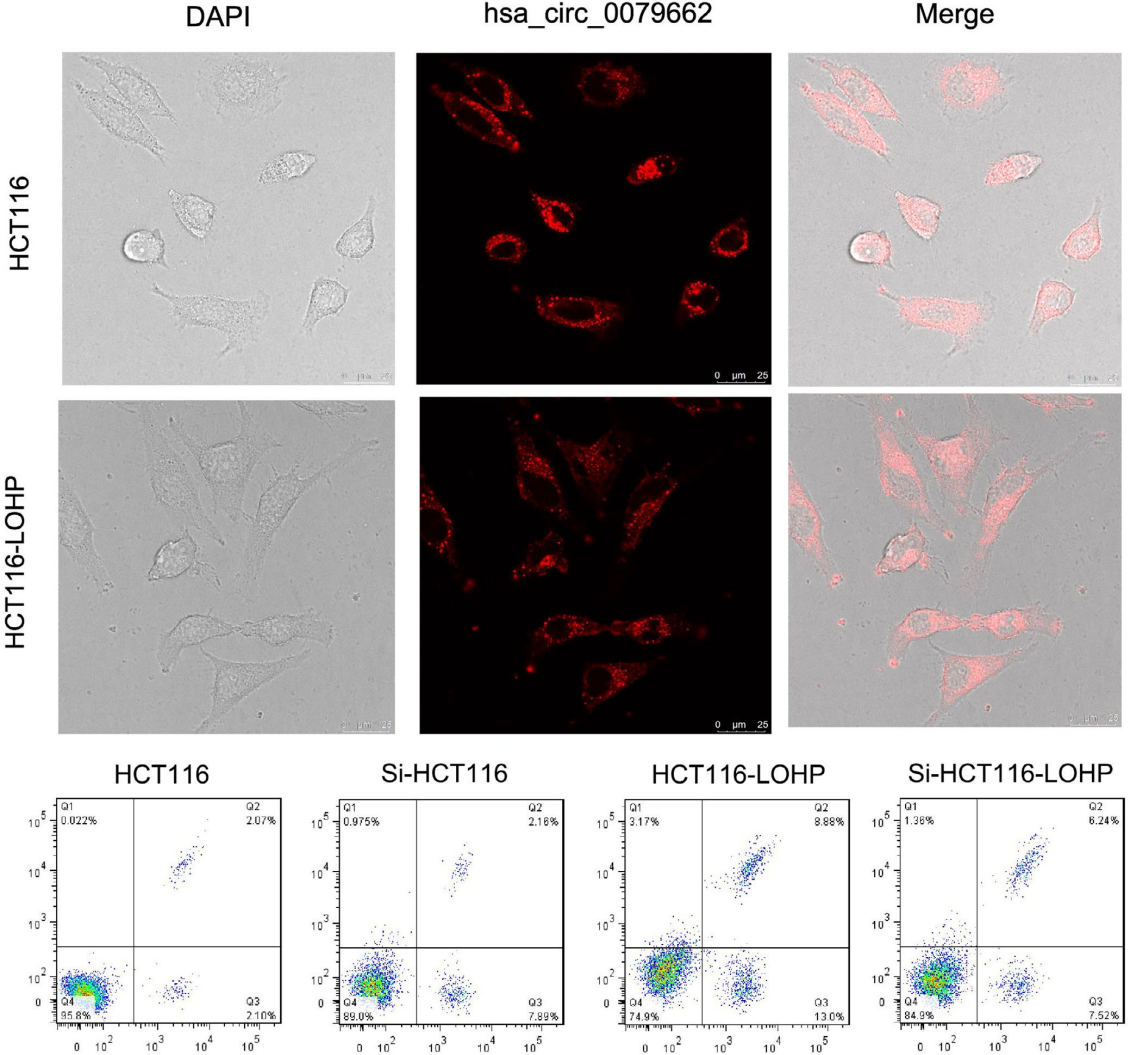
Hsa_circ_0079662 promotes growth of drug‐resistant CRC cell lines

Having known hsa_circ_0079662 was up‐regulated in A1, A2 and A3. We then explored the onco‐genic properties and roles of hsa_circ_0079662. We established cell lines with hsa_circ_0079662 stable overexpression. Results demonstrated silence of hsa_circ_0079662 induced a reduction in the cell growth than that of in their blank counterparts (Figure 2). Overexpression of hsa_circ_0079662 showed increase in the cell growth (Figure 2). These results clearly demonstrated that hsa_circ_0079662 significantly facilitated cell growth in CRC cells.

### 3.3 Hsa_circ_0079662 drug‐resistant roles are partially via spongeing hsa‐mir‐324‐5p, and then activating HOXA9

**Figure 3:**
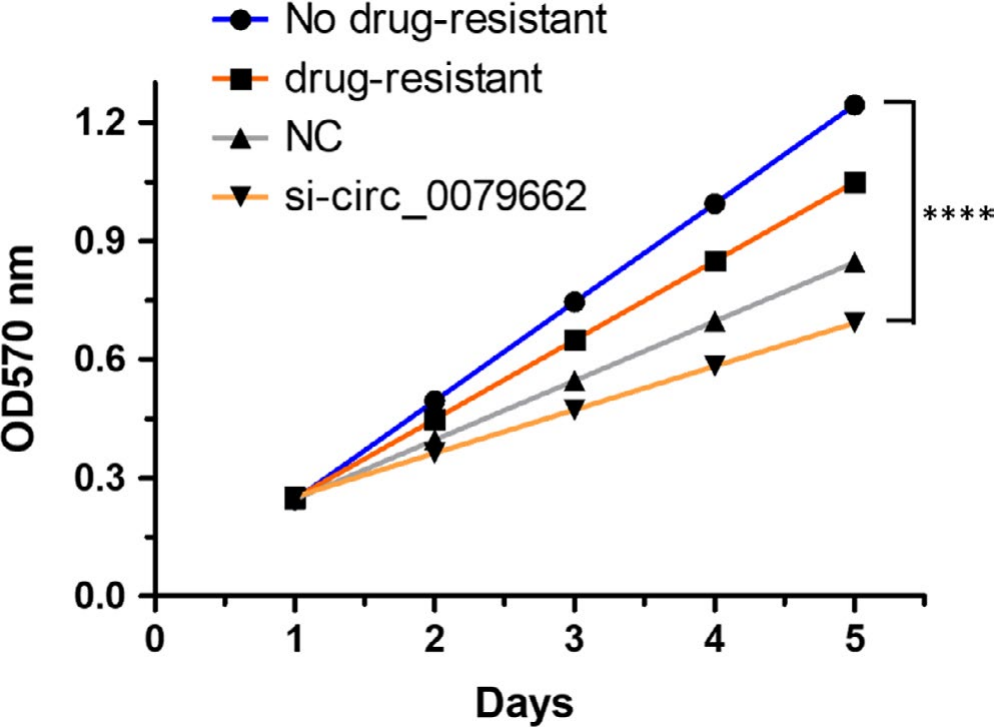
Hsa_circ_0079662 in hsa‐mir‐324‐5p treated cells, significantly reversed the growth‐inhibitory role. ****P < .001

**Figure 4:**
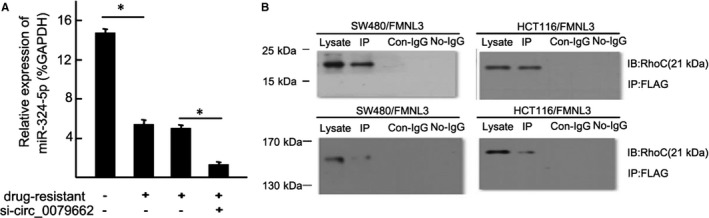
Hsa‐mir‐324‐5p treatment decreased cell growth of cells. *P < .05

**Figure 5:**
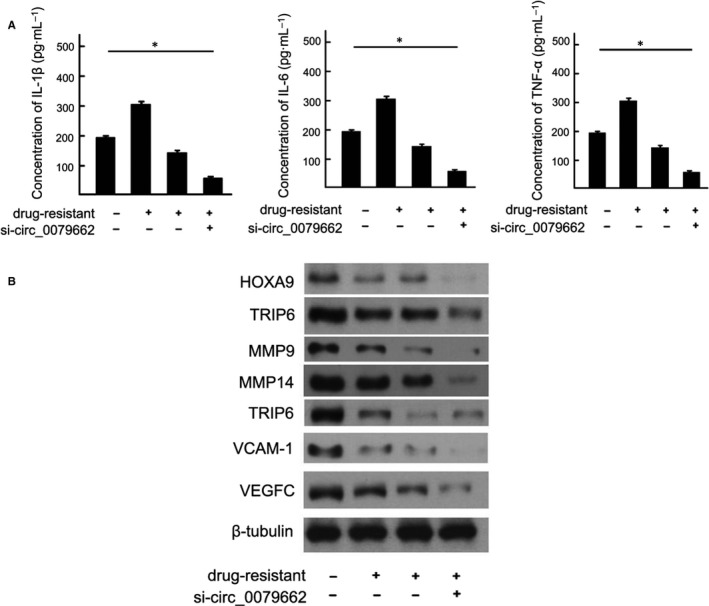
The expression of cytokines TNF‐α, IL‐1 and IL‐6 was increased in drug‐resistant CRC. *P < .05

Hsa‐mir‐324‐5p repressed cell growth in CRC cell lines, while overexpressed hsa_circ_0079662 in has‐mir‐324‐5p treated cells, significantly reversed the growth‐inhibitory role (Figure 3). We found a well‐known oncogene, HOXA9, was up‐regulated, which could be a direct target of hsa‐mir‐324‐5p. Results demonstrated hsa‐mir‐324‐5p treatment decreased cell growth of cells (Figure 4). Results revealed that hsa‐mir‐324‐5p and si‐HOXA9 treatment inhibited expression of HOXA9, while hsa_circ_0079662 treatment significantly increased protein expression in cells (Figure 4). Our results reveal that hsa‐mir‐324‐5p targets human HOXA9 by directly binding to the predicted sites in 3′‐UTR of HOXA9 mRNA. Furthermore, the expression of HOXA9, TRIP6, Vcam‐1, VEGFC, MMP3, MMP9 and MMP14 was higher, and the expression of cytokines TNF‐α, IL‐1 and IL‐6 was increased in drug‐resistant CRC (Figure 5).

## 4 DISCUSSION

CRC is the most common colon cancer in Western Europe, North America and other developed countries, and also one of the nine colon cancers in China. Over the past 30 years, the incidence of colon cancer has been on the rise in most countries and regions. Colon cancer is the fifth leading cause of death among men and the sixth leading cause of death among women. From the viewpoint of epidemiology, colonic cancer come on with social environment, lifestyle (especially dietary habit, lack physical activity), genetic element is concerned. Age, colorectal polyps, ulcerative colitis and cholecystectomy are also risk factors for colon cancer. But overall, the cause of colon cancer seems unclear.^30^, ^31^ This study is the first to investigate the biological roles of hsa_circ_000984 in CRC carcinogenesis.

In cytoplasm, circRNA sequence also contains MREs (miRNA response elements) as ceRNA which ACTS as a sponge to adsorb miRNA, thus regulating the abundance of target genes.^32^, ^33^, ^34^ Since circRNA has no poly(A) tail and 5′ terminal, it is more difficult to be degraded than linear RNA molecules, so it has the advantage of being miRNA sponge. At present, more and more studies have shown that circRNA can play the role of miRNA sponge and participate in tumour proliferation, invasion, metastasis, apoptosis and angiogenesis.^35^, ^36^, ^37^, ^38^ Different from the traditional linear RNA (containing 5′ and 3′ terminal), circRNA molecules have a closed ring structure and are not affected by RNA exonuclease, so their expression is more stable and not easily degraded. In terms of function, recent studies have shown that circRNA molecules are rich in miRNA binding sites, which can act as miRNA sponge in cells, and then remove the inhibition effect of miRNA on its target genes and increase the expression level of the target genes. This mechanism is called the ceRNA mechanism.^39^, ^40^, ^41^, ^42^ Through the interaction of miRNAs associated with diseases, circRNAs play an important regulatory role in diseases. In this study, we found that HOXA9 was an important component of the hsa_circ_0079662/hsa‐miR‐324‐5p network. Based on RT‐PCR, Western blotting, and Transwell assays, we found that HOXA9 was repressed by has‐miR‐324‐5p.

In this present study, we found that hsa_circ_0079662 was significantly up‐regulated in CRC. The knock‐down of hsa_circ_0079662 inhibited the proliferation, migration, invasion of CRC cells in vitro and in vivo by our series of experiments. CircRNA plays a strong regulatory function in carcinoma by acting as a sponge of miRNAs.^42^, ^43^ We first found that hsa_circ_0079662 might interact with hsa‐miR‐324‐5p, which can bind to hsa‐miR‐324‐5p as a miRNA sponge. Dysregulated activation of the HOXA9 is a hallmark of a variety of carcinomas.^43^

In summary, the expression of has_circ_0079662 is up‐regulated in CRC cell. Hsa_circ_0079662 affected CRC cell growth, migration and invasion by binding with hsa‐miR‐324‐5p. The hsa‐miR‐324‐5p may be as a potential molecular markers for promising application perspectives of CRC.

## CONFLICT OF INTEREST

The authors declare no conflict of interest.

## AUTHOR CONTRIBUTION

Jiazhuan Mei wrote the manuscript and Mingfen Lai, Guiju Liu, Ruijun Li, Hua Bai conducted most of the experiments, Jizhi Zhao, Peng Xiao collected the data, Ruijun Li, Hua Bai, Jizhi Zhao analysed the data, Mingfen Lai, Jiazhuan Mei designed the study and all authors approved the submission.

## Data Availability

Data can be obtained from the corresponding author on request.
